# Supplementary material for: Dietary Exposure to 2,2′,4,4′-Tetrabromodiphenyl Ether (BDE-47) Causes Inflammation in the Liver of Common Carp (Cyprinus carpio) and Affects Lipid Metabolism by Interfering with Steroid Hormone Biosynthesis Pathways
Source: Int J Mol Sci. 2025 Oct 18;26(20):10152. doi: 10.3390/ijms262010152 (PMC12563177; doi:10.3390/ijms262010152)

Supplementary

Table S1. Gene-special primers used in qPCR

Gene	Forward 5′to'3	Reverse 5′to'3
ACC1	GTCACTGGCGTATGAGGATATT	TCCACCTGTATGGTTCTTTGG
FAS	GTGTACGCCACCGCCTATTA	ATAGCAATAGCGGCCTGTCC
RXRα	CCGCAACGAGAACGAGGTG	AGGGCATCGGGACATTGGT
SREBP1	CGTCTGCTTCACTTCACTACTC	GGACCAGTCTTCATCCACAAA
atgl	CTCCACCAACATTCACGAGC	CTCTGTATCCCTGCTTGCAC
hsl F	ATGATTTGGATGCGCAGACC	AAACGCTCCAGTGCAGTTTG
lpl F	CGCTCCATTCACCTGTTCAT	GCTGAGACACATGCCCTTATT
star	GTGGAACCCCAATGTCAAAC	ACAGGTGGGTCCATTCTCAG
11β-hsd3	GCACTCAATGGTTTCTTTGGA	GCTCCAGCTTCGATAATGTG
3βHSD	CTGAGAGGAGAGGTGTGTGTGTGTGT	GTTTGTCAGTCGGATCTCGG
tnf- α	TCGCCGGACTTCACAATAGG	GCTTGCTCGCCAGTTGTTTT
tgf- β 1	ATTCAGAACTATAAGACCCCCC	CGGAAGTCAATGTAAAGAGGC
il-1 β	TGATGAACGAGCTGGATGGG	GCTGGGTCTGCGGTATGTAG
il-10	TTGTGGTCTGTCTCAGTCGTG	TGTTCTTGAAAGCCCTCCT
β-actin	GATGGACTCTGGTGATGGTGTGAC	TTTCTCTTTCGGCTGTGGTGGTG
gadph	TGTGGGCATCAATGGATTTGG	ACACCATGTATTCCGGGTCAAT

Figure S1. Gene expression of factors related to lipid metabolism. Expression of acc (A)*, fas* (B)*, rxrα* (C)*, srebp* (D)*, atgl* (E)*, hsl* (F)*, lpl* (G)*,star* (H), *hsd3β* (I)*, 11βhsd* (J)*, pi3k* (K)*, akt* (L) *and pparγ* (M). All data are presented as mean ± standard error of measurement (SEM), and different letters (a-c) indicate significant differences. The letters from a to c represent significance from low to high, and if different groups have the same letter, it means that there is no significant difference between the two groups.

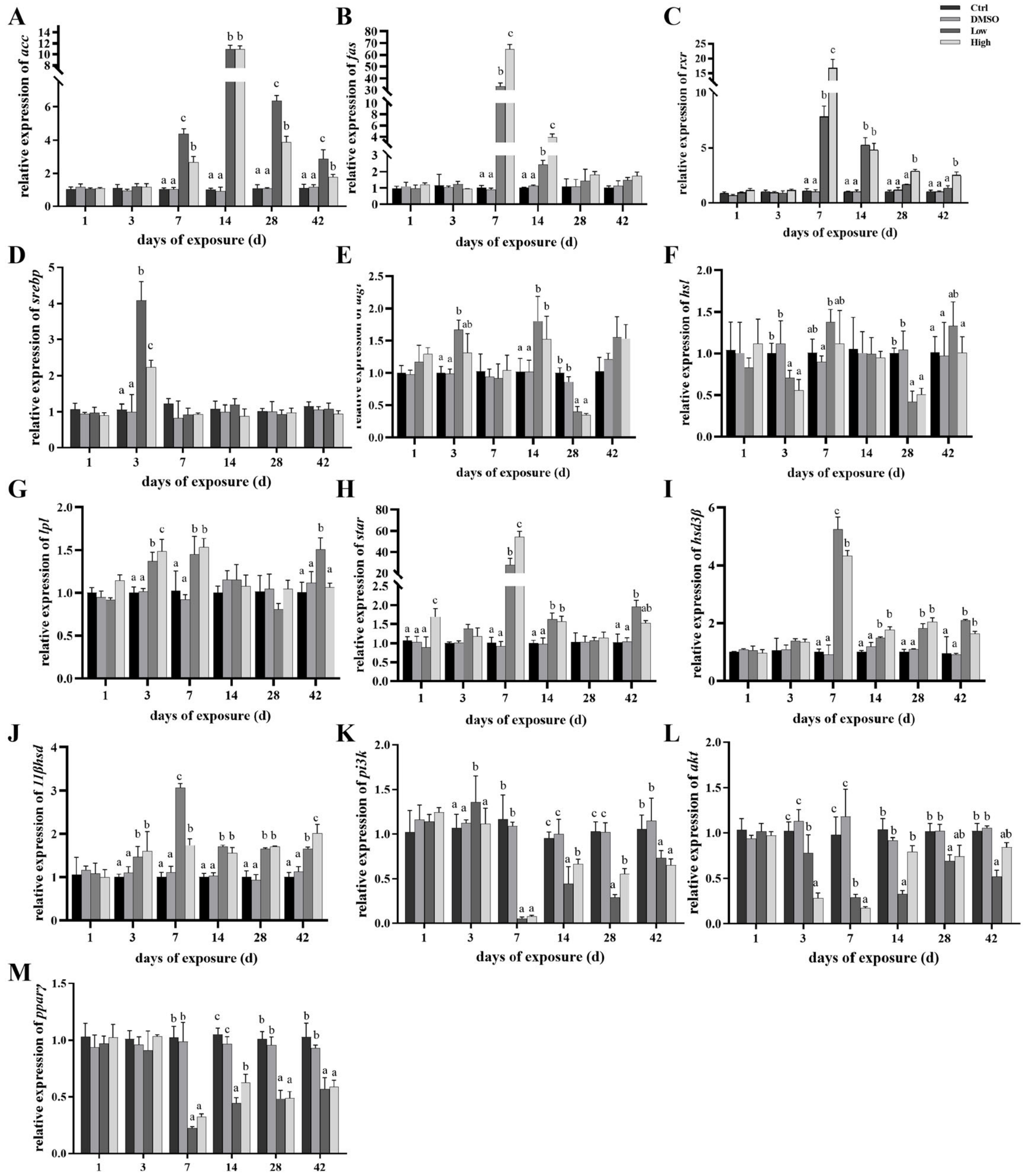

Figure S2. PCA analysis between different treatment groups (A), heatmap of correlation coefficients between IDO and GLU, TG, CHO, and GLYC (B), orange indicates positive correlation, green indicates negative correlation, and the deeper the color, the greater the absolute value of the number, the stronger the correlation.

A

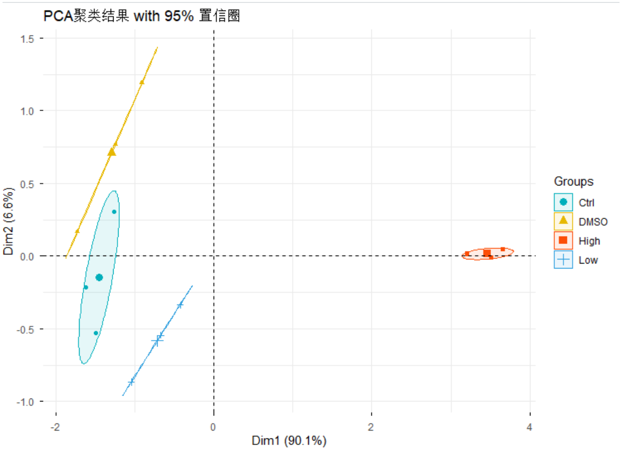

B

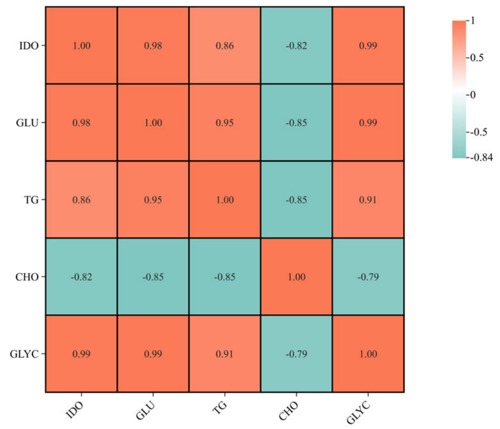

Supplement: Supplementary file 1 [file ijms-26-10152-s001.zip › ijms-3855075-supplementary.pdf]
